# Treatment Patterns and Healthcare Outcomes with Collagenase Clostridium Histolyticum vs Surgery in Peyronie's Disease: A Retrospective Claims Database Analysis

**DOI:** 10.1016/j.esxm.2021.100321

**Published:** 2021-03-05

**Authors:** Landon Trost, Huan Huang, Xu Han, Chakkarin Burudpakdee, Yiqun Hu

**Affiliations:** 1 Department of Urology, Mayo Clinic, Rochester, MN, USA; 2 Male Fertility and Peyronie's Clinic, Orem, UT, USA; 3 IQVIA, Falls Church, VA, USA; 4 Endo Pharmaceuticals Inc, Malvern, PA, USA

**Keywords:** Collagenase Clostridium Histolyticum, Penile Complications, Peyronie's Disease, Treatment Patterns, Treatment Outcomes

## Abstract

**Introduction:**

Treatments for Peyronie's disease (PD) include surgical management and collagenase clostridium histolyticum (CCH).

**Aims:**

To evaluate PD treatment trends after CCH approval and compare clinical outcomes in CCH- and surgery-treated cohorts.

**Methods:**

Patients newly diagnosed with PD between January 2011 and December 2017 were identified in a U.S. claims database. Cohorts initiating treatment with CCH or surgery between January 2014 and June 2017 were included. Patients were continuously enrolled ≥6 months before and ≥12 months after index date. Post-treatment penile complications and analgesic use were compared 1 year after procedure in propensity score-matched cohorts.

**Main outcome measures:**

The main outcome measures of this study were treatment patterns, penile complications, and analgesic use.

**Results:**

In the newly diagnosed PD cohort, 1,609 patients received CCH and 1,555 patients had surgery. Overall CCH or surgery treatment rate/year increased from 9.8% in 2014 to 15.5% in 2017, with <1% receiving verapamil or interferon. Initial treatment ratios of CCH to surgery increased from approximately 1:1 (2014) to 2:1 (2017). In the unmatched CCH (n = 1,227) and surgery (n = 620) cohorts, more (*P* < .05) surgery-treated patients received analgesics (particularly opioids), oral PD therapies, vacuum erection devices, and phosphodiesterase-5 inhibitors before the index date. After propensity score matching (n = 620/cohort), newly occurring postprocedural complications during the follow-up period were higher in the surgery cohort (25.3% vs 18.4%, *P* = .003). The surgery cohort had significantly (*P* < .05) higher rates of erectile dysfunction (65.0% vs 44.8%), penile pain (17.9% vs 8.9%), and penile swelling (8.1% vs 5.2%) and was more likely to be prescribed opioids (93.3% vs 38.9%; *P* < .0001) or non-steroidal anti-inflammatory drugs (27.0% vs 20.3%; *P* = .006).

**Conclusion:**

CCH demonstrated fewer complications and less analgesic use than surgery and was used as the initial therapy for PD twice as often as surgery.

**L Trost, H Huang, X Han, et al. Treatment Patterns and Healthcare Outcomes with Collagenase Clostridium Histolyticum vs Surgery in Peyronie's Disease: A Retrospective Claims Database Analysis. Sex Med 2021;9:100321.**

## Introduction

Peyronie's disease (PD) is characterized by a disorganized, excessive deposition of collagen that results in plaque formation within the penile tunica albuginea.^1^^,^^2^ This plaque formation can restrict tunica lengthening during penile erection, resulting in penile curvature, deformity, discomfort, and/or pain.^1^ The reported U.S. prevalence of PD ranges from 0.5% to 13%, although this may be underestimated owing to a reluctance of men to admit to the condition and seek treatment for it.^2^^,^^3^ Although PD occurs predominantly in older men, it has been reported in nearly every age group.^3^, ^4^, ^5^, ^6^

Treatment goals are to maximize symptom control, sexual function, and patient/partner quality of life while minimizing adverse events and patient/partner burden.^1^ Historically, surgical management of PD was considered the “gold standard” treatment for men with intact erectile function and PD, with multiple variations of plication, corporoplasty, or incision/excision and grafting techniques described.^1^^,^^7^ However, surgery is associated with complications including penile length/volume loss, erectile dysfunction (ED), sensory changes, recurrence of curvature, and palpable abnormalities, among others.^8^ Given a desire for more conservative therapies, other treatments, including oral and topical formulations, have been proposed; however, limited, conflicting data have failed to consistently show benefits.^1^^,^^9^

In December 2013, the U.S. Food and Drug Administration approved the first injectable therapy for the treatment of PD (collagenase clostridium histolyticum [CCH] [Xiaflex; Endo Pharmaceuticals Inc, Malvern, PA]), based on 2 phase III, randomized clinical trials that demonstrated safety and efficacy in treating penile curvature.^10^^,^^11^ Since then, multiple postapproval studies have confirmed the clinical utility and efficacy of CCH in various PD cohorts.^12^, ^13^, ^14^ However, limited data are currently available on utilization rates of CCH and changes in practice patterns that have occurred since its availability. A retrospective regional claims database analysis of patients with PD between 2013 and 2016 showed that use of injectable therapies, including CCH, is increasingly displacing surgical management as first-line treatment in clinical practice.^15^

The current objectives were to review a nationally representative healthcare claims database to describe therapeutic trends in PD treatment in newly diagnosed patients after U.S. regulatory approval of CCH for PD and to compare clinical outcomes of men treated with CCH vs surgery. The study hypothesis was that after approval and release, CCH use would increase and surpass surgery as a first-line treatment for PD.

## Materials and methods

### Study Design

This retrospective, longitudinal cohort study was conducted using administrative healthcare claims data gathered from the IQVIA Real-World Data Adjudicated Claims, a U.S. database, between January 1, 2010 and June 30, 2018. This database contains anonymized information for >150 million unique enrollees representing a diverse cohort by U.S. geographic region (ie, northeast, midwest, south, west), employers, payers (eg, commercial, Medicaid, Medicare, self-insured), providers, and specialists. The data collected included demographic variables and medical and prescription claims data (eg, inpatient and outpatient diagnoses, procedures, and medications). The database is considered to be representative of the U.S. insured population with regard to age and sex.^16^ This database is further described by Camper et al (2019).^16^

Three patient cohorts were created for this study: 1 cohort for the treatment trend analysis and 2 cohorts for comparing patient characteristics, treatment patterns, post-treatment penile complications, and analgesic use (ie, non-steroidal anti-inflammatory drugs, opioids) among patients initiating CCH vs surgery. For the treatment trend analysis, a cohort that was newly diagnosed with PD between January 2011 and December 2017 was created (Supplementary Figure 1). To ensure that patients were newly diagnosed, those with a PD diagnosis recorded during the 12 months before the first observed PD diagnosis were excluded. Continuous enrollment ≥12 months before the first PD diagnosis was required to ensure an adequate look-back period. Continuous enrollment for ≥30 days after the first PD diagnosis was required to capture treatment trends. The first treatment received (ie, CCH, penile plication, incision/excision and grafting, penile prosthesis, interferon alpha, or verapamil) and the time from diagnosis to the first treatment were assessed. In cases where men underwent >1 treatment during the study period, only the first treatment was captured. Patients with PD were identified based on diagnosis codes (International Classification of Diseases, Ninth/Tenth Revision, Clinical Modification), and treatment of interest was identified based on National Drug Codes, the Healthcare Common Procedure Coding System, or Current Procedural Terminology codes (Supplementary Tables 1–4).

**Figure 1:**
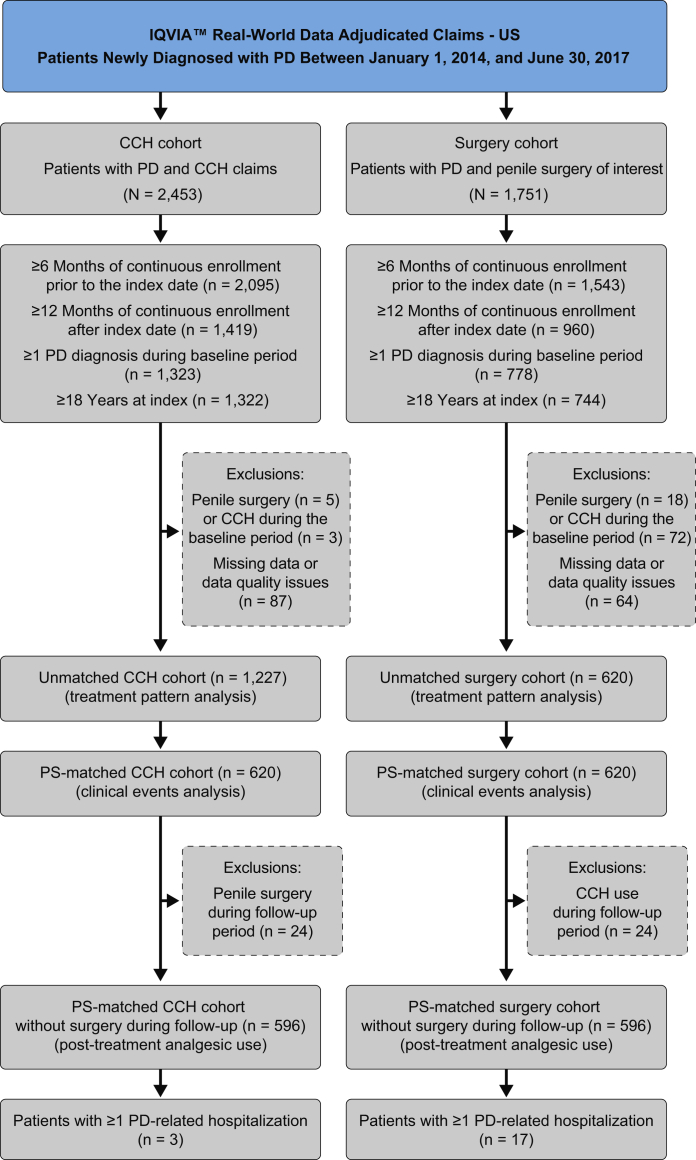
Unmatched and PS-matched CCH and surgery cohorts. CCH = collagenase clostridium histolyticum; PD = Peyronie's disease; PS = propensity score.

For the comparative analysis, the same database was used to create the cohorts that included patients initiating intralesional CCH therapy or penile surgery of interest (ie, plication, incision/excision and grafting, and penile prosthesis implantation) between January 2014 and June 2017 (Figure 1). This selection period was chosen to coincide with the approval of CCH. The index date for the CCH cohort was defined as the date of the first CCH claim; for the surgery cohort, it was defined as the date of the first surgery of interest (Supplementary Figure 2). Patients were included if they were ≥18 years of age on the index date and had continuous enrollment of ≥6 months before the index date (baseline), to capture prior PD therapies, and ≥12 months after index (follow-up period). Patients were required to have ≥1 medical claim with a PD diagnosis and no evidence of penile surgery of interest or CCH treatment during the baseline period. The CCH cohort was matched 1:1 to the surgery cohort using propensity score (PS) matching based on baseline characteristics (ie, age category, Charlson comorbidity index [CCI] category, comorbidities, geographic region, history of radical prostatectomy, indicator of ≥1 all-cause hospitalization, insurance plan type, payer type, total all-cause cost/month per patient, treatment of PD). Outcomes, including penile-related complications and analgesic use, were compared between PS-matched cohorts during the 12-month postindex period.

### Study Assessments

For the treatment trend analysis, the number of patients newly diagnosed with PD, the initial treatments received, and time from the diagnosis to initial treatment were analyzed. For the comparative analysis, baseline demographic and clinical characteristics and penile events of interest were compared before and after PS matching (Supplementary Tables 4 and 5). Time from earliest PD diagnosis to the initial treatment with CCH or surgery (index event) and PD-related treatments used during the follow-up period (eg, analgesics, intralesional injections) were also evaluated, as were postindex penile-related complications and medication use. For the CCH cohort, the total number of CCH injections (identified using Healthcare Common Procedure Coding System codes) per patient was evaluated.

### Statistical Analysis

All measures were reported with descriptive statistics, using frequencies and percentages for categorical variables and mean, SD, median and interquartile ranges (Q1–Q3) for continuous variables. For the comparative analysis, baseline patient characteristics of the unmatched CCH and surgery cohorts during the preindex period were described. The *t*-test (mean) and Wilcoxon rank-sum test (median) were used to compare continuous variables between the unmatched cohorts, while χ^2^ tests were used for categorical variables. All tests were conducted assuming a two-tailed test of significance and α = .05.

The CCH and surgery cohorts were compared after PS matching to minimize confounding and bias. A PS model that estimated the probability of receiving either CCH or surgery was constructed from key baseline characteristics including: duration of preindex period, index age group, geographic region, payer type, plan type, CCI categories,^17^ selected comorbidities (including benign prostatic hyperplasia, hypogonadism, diabetes, cardiovascular disease, dyslipidemia, Dupuytren's contracture, lower urinary tract symptoms, prostate cancer, depression, obesity, ED, penile pain), and history of radical prostatectomy.

Postprocedural treatment outcomes, including penile complications and analgesic use during postindex period, were compared using the PS-matched cohorts. Pair-wise comparisons were made between PS-matched cohorts using the Wilcoxon signed-rank test for continuous variables and the McNemar-Bowker test for categorical variables. Statistical analyses were performed using SAS software, version 9.4 (SAS Institute, Inc, Cary, NC).

## Results

### Treatment Trends

**Figure 2:**
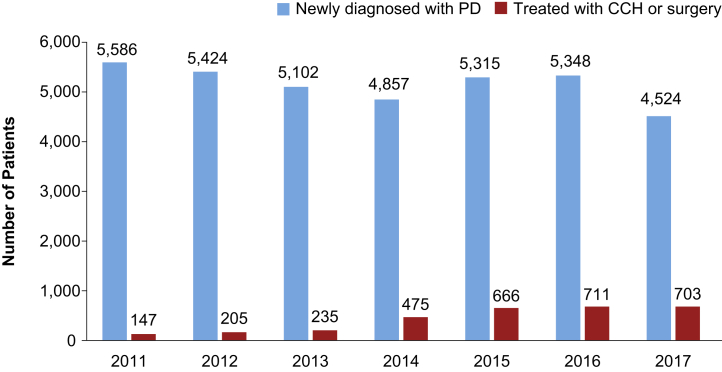
Number of newly diagnosed and newly treated (initial treatment) patients with PD by year. CCH = collagenase clostridium histolyticum; PD = Peyronie's disease.

**Figure 3:**
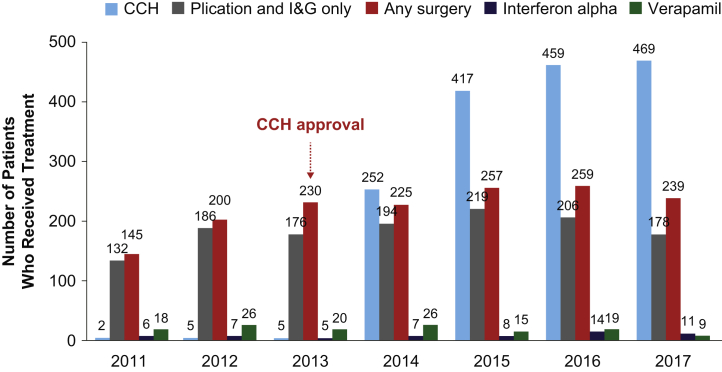
Comparative use of PD treatments by year in newly diagnosed patients. CCH = collagenase clostridium histolyticum; I&G = incision and grafting; PD = Peyronie's disease.

In the cohort of patients newly diagnosed with PD (n = 36,156) identified between 2011 and 2017, 1,555 patients had surgery and 1,609 received CCH as initial therapy (Supplementary Figure 1). During this time period, while the annual rate of new PD diagnoses remained stable, the treatment rate with CCH or surgery increased gradually, from 9.8% in 2014 to 15.5% in 2017 (Figure 2). After the release of CCH in 2014, its use as first-line treatment for PD increased by 1.6%/year (*P* = .023 for yearly trend), whereas the rate for surgery remained stable (0.2%/year, *P* = .078). The ratio of CCH vs surgery as initial treatment for PD increased from approximately 1:1 in 2014 to approximately 2:1 in 2017 (Figure 3). When stratifying this newly diagnosed cohort by surgery type, the percentage of patients treated with plication and incision and grafting decreased by 18.8% in 2017 from its peak in 2015, while the percentage of patients receiving a penile prosthesis increased by 11.9% from 2015 to 2017.

**Figure 4:**
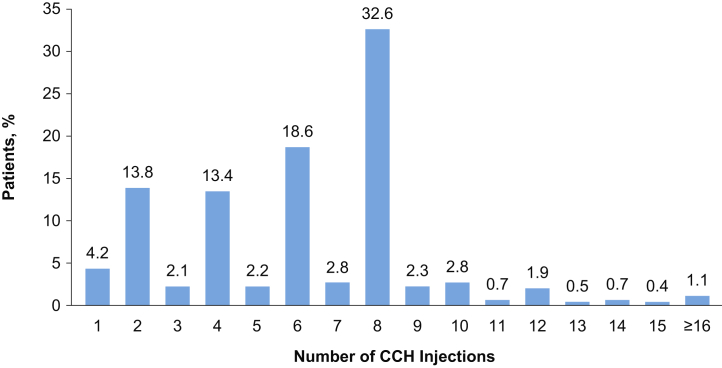
Distribution of the number of CCH injections (N = 1,044). CCH = collagenase clostridium histolyticum.

Between 2014 and 2017, the mean time from the initial PD diagnosis to the first CCH treatment decreased from 13 months in 2014 to 8 months in 2017. The mean time from diagnosis to first treatment also decreased in the surgery cohort, although at a slower rate that was not statistically significant (0.7 vs 1.8 months/year, *P* = .13). The use of other intralesional injection therapies (eg, verapamil or interferon) as the initial treatment remained consistently low (<1%); however, these therapies often are not submitted for insurance claims and may be underrepresented in the current cohort. Patients treated with CCH received a median of 6 injections; the overall distribution of injections indicated that 32.6% of patients received 8 injections (Figure 4).

### Comparative Analysis

#### Baseline Patient Demographics, Clinical Characteristics, Treatment History

There were 1,227 CCH cohort and 620 surgery cohort patients identified from 2014 to 2017 before PS matching. During the baseline period, there were statistically significant differences between both unmatched cohorts in age group at index, mean CCI score, select comorbidities, analgesic use, prior PD therapies, and prior ED therapies (Table 1, Supplementary Tables 5 and 6).

**Table 1:** Baseline demographic characteristics before and after PS matching

Measures	Before PS matching			After PS matching		
	CCH (N = 1,227)	Surgery (N = 620)	*P* value	CCH (N = 620)	Surgery (N = 620)	*P* value
Duration of baseline period (mo)						
Mean (SD)	46.4 (25.0)	43.3 (24.1)	.011	43.4 (24.3)	43.3 (24.1)	.911
Median (IQR)	50.6 (22.7, 67.3)	43.0 (20.9, 63.1)	.008	43.2 (20.0, 62.6)	43.0 (20.9, 63.1)	.983
Min	6.0	6.0		6.0	6.0	
Max	91.1	89.0		91.1	89.0	
Age (y)						
Mean (SD)	54.7 (7.3)	54.2 (9.0)	.159	54.5 (7.8)	54.2 (9.0)	.490
Median (IQR)	56 (51, 60)	56 (51, 60)	.901	56 (51, 60)	56 (51, 60)	.970
Min	18	18		18	18	
Max	72	72		70	72	
Categories			.034∗			.889∗
18–34	25 (2.0%)	28 (4.5%)	ND	22 (3.5%)	28 (4.5%)	ND
35–44	78 (6.4%)	36 (5.8%)	ND	37 (6.0%)	36 (5.8%)	ND
45–54	405 (33.0%)	185 (29.8%)	ND	193 (31.1%)	185 (29.8%)	ND
55–65+	719 (58.6%)	371 (59.8%)	ND	368 (59.4%)	371 (59.8%)	ND

CCH = collagenase clostridium histolyticum; IQR = interquartile range; ND = not determined; PS = propensity score.

∗ To detect statistical differences in age distribution between the cohorts, all age categories were compared.

After PS matching, 620 patients remained in each cohort. The matched cohorts showed similar baseline demographic characteristics (mean age of 54 years at index) and similar clinical characteristics (Table 1, Supplementary Table 5). The mean CCI score was approximately 1.5 for both cohorts; the most common comorbidities being ED, cardiovascular diseases, dyslipidemia, and benign prostatic hyperplasia. In the matched cohorts, there was a higher percentage of patients with a history of radical prostatectomy in the surgery cohort than in the CCH cohort (5.2% vs 1.9%, *P* = .001). The majority of patients in the matched CCH (73.9%) and surgery (76.5%) cohorts filled prescriptions for analgesics (ie, opioid or non-steroidal anti-inflammatory drugs [NSAIDs]) at some point before their index treatment (Supplementary Table 6). CCH and surgery cohorts were similar in their use of oral and intralesional PD therapies and ED treatments such as phosphodiesterase-5 inhibitors and testosterone.

#### Postprocedural Complications

**Figure 5:**
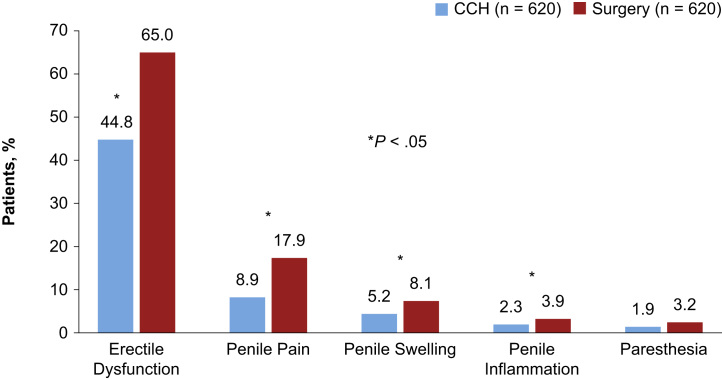
Penile-related events during the 12-month postindex period (PS-matched cohorts). CCH = collagenase clostridium histolyticum; PS = propensity score.

During the 12-month follow-up period (PS-matched cohorts), the surgery cohort had a higher percentage of patients with newly occurring postprocedural complications vs the CCH cohort (25.3% vs 18.4%, *P* = .003). Among all postprocedural penile-related complications (newly occurring and reoccurring), several notable complications occurred at significantly higher rates in the surgery vs CCH cohort, including ED (65.0% vs 44.8%), penile pain (17.9% vs 8.9%), and penile swelling (8.1% vs 5.2%; Figure 5). In contrast, corporal rupture (1.8% vs 0.8%) and penile hematoma (1.1% vs 0.2%) were reported more frequently in men treated with CCH, although differences for the latter 2 comparisons were not statistically significant (Supplementary Table 7).

#### Postprocedural Analgesic Use and Hospitalization

**Figure 6:**
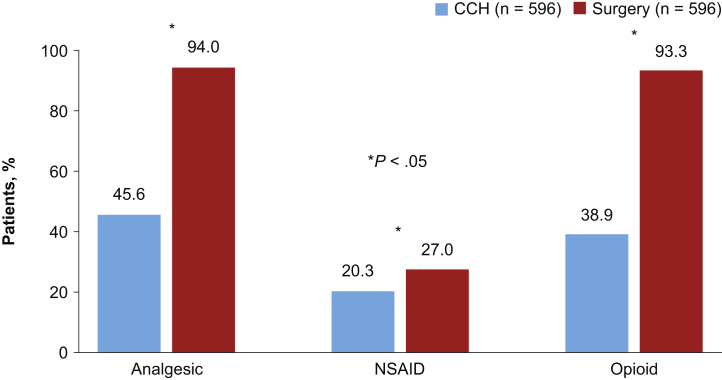
Analgesic use during the 12-month postindex period (PS-matched cohorts). CCH = collagenase clostridium histolyticum; NSAID = non-steroidal anti-inflammatory drug; PS = propensity score.

Analgesic use and hospitalization rates were evaluated in the PS-matched cohorts. To limit confounding, patients in the CCH cohort who subsequently underwent surgery, and patients in the surgery cohort who subsequently received CCH were excluded, resulting in a total of 596 patients remaining in each cohort (Figure 1). Within 12 months before the index date, 38.6% of patients in the CCH cohort and 43.5% of patients in the surgery cohort filled at least 1 opioid prescription. During the 1-year postindex follow-up period, the use of opioids (93.3% vs 38.9%; *P* < .0001) and NSAIDs (27.0% vs 20.3%; *P* = .006) was higher in the surgery cohort than in the CCH cohort (Figure 6). The mean (SD) number of opioid prescriptions per patient was also higher in the surgery vs CCH cohort (4.4 [5.7] vs 1.8 [4.1]; *P* < .0001), and nearly all opioid prescriptions (94.8%) were filled within the first week after the surgical date. Patients receiving surgery were more likely than patients treated with CCH to be hospitalized for PD-related complications during the follow-up period (2.9% vs 0.5%; *P* = .002).

## Discussion

This is the first study to report findings on observed treatment patterns and outcomes in the U.S. commercially insured, newly diagnosed, and newly treated PD population. Findings demonstrated that CCH is more commonly used as initial therapy for PD and that its use as first-line treatment doubled over surgery by 2017. In addition, results showed that an increasing number of men sought treatment with either CCH or surgery overall, suggesting that the option of an effective, conservative therapy led more men to seek treatment for PD. The time from diagnosis to initial treatment has also decreased throughout the observed period, which may indicate that patients are seeking out treatment earlier or that providers are offering CCH sooner than previously offered. The findings from the present study are consistent with other published data. Sukumar et al^15^ (2019) reported an analysis of CCH claims in New York state during a similar time period. In their series, CCH was used as the first-line therapy for most patients newly diagnosed with PD over surgery, and CCH was used more frequently than surgery as a treatment option.

The specific reasons for the preferential use of CCH as a first-line therapy were not directly captured in the present study; however, the minimally invasive nature of CCH and lower postindex complication rates compared with surgery, as demonstrated in this study, may be contributing factors. Results from the present study highlight a lower postprocedural complication rate in the CCH (18.4%) vs surgery cohort (25.3%; *P* = .003). Although rare, patients treated with a surgical procedure were also more likely to be hospitalized for a procedure-related complication than those treated with CCH. A unique finding of this study was the frequency of opioid use in the CCH and surgery cohorts before and after the procedures. During the 12 months before the index date, approximately 40% of men with PD had filled a prescription for opioids. Twelve months after the index date, the percentage of patients who filled opioid prescriptions was 2.4-fold higher in the surgery cohort than the CCH cohort (93.3% vs 38.9%; *P* < .0001), with almost all prescriptions in the surgery cohort filled within a week of the procedure.

The present study has several limitations that are inherent to claims-based methodologies, including potential misclassification of events based on diagnosis codes. In addition, the present study population was only a sample of all patients with PD in the United States. However, despite the small absolute numbers, the relative findings can be generalized to other U.S. commercially insured patients, given the broad sampling across the country.^16^ Inclusion of patients receiving penile prostheses in the surgery cohort is a limitation, given that this subgroup of patients likely represents a distinct population that may experience greater rates of postoperative pain and hospitalizations compared with groups of patients receiving other interventions. The frequencies of treatments reported during the study period likely underestimates the total number of surgical procedures and CCH injections performed annually. Based on Endo Pharmaceutical's internal data, approximately 37,500 vials of CCH were distributed from specialty pharmacies for PD use in 2017. Using a mean treatment of 6 vials per patient, this would suggest that approximately 6,250 unique patients with PD would have received treatment with CCH in 2017. This notably contrasts with the lower rate identified in the present study (n = 1,609). There are likely several reasons for this discrepancy, including evaluation of only men with newly diagnosed PD, extraction of only the first postdiagnosis treatment, specific requirements for look-back and look-forward periods, limited inclusion of Medicare/Medicaid beneficiaries, and other data quality requirements. However, despite these limitations, the data for the 2 treatment modalities are likely the most accurate as what can be reliably captured in a methodologically sound manner and provide insights from a comparative standpoint. For instance, the ratio of incremental CCH use reflected in the trending analysis in this study was consistent with CCH specialty pharmacy data, showing a 230% increase in the number of CCH vials distributed in 2017 compared with 2014.

Another limitation of insurance claims databases is inability to assess disease course and severity (eg, pretreatment disease history, degree of penile curvature), reasons underlying treatment selection (eg, insurance coverage of specific therapies), or patient-reported outcomes (eg, patient satisfaction). There was also limited representation of Medicare and Medicaid beneficiaries, and therefore, findings may not be generalizable to the uninsured, underinsured, and patients 65 years and older. Finally, some treatments used to manage PD (eg, NSAIDs, omega-3-fatty acids, traction, interferon alpha, verapamil) are available over the counter and/or are not submitted to insurance carriers for reimbursement; therefore, their use may be underestimated in the present study. In addition, although the number of patients filling a prescription was captured in the database, actual utilization was not tracked.

Despite these limitations, the current data set is the largest of its kind and presents a nationally representative report of how trends have changed in PD management since the introduction of CCH. The data additionally demonstrate an increasing use of CCH as first-line therapy for PD and are the first to report actual utilization rates of CCH and compare different complication rates between CCH and surgery in a scientifically rigorous manner.

## Conclusions

Results of this retrospective claims database analysis demonstrate increasing use of CCH vs surgery as first-line treatment for newly diagnosed PD in the real-world clinical practice setting. Compared with surgery, CCH treatment of PD was associated with lower rates of penile-related complications, hospitalization for PD-related complications, and use of opioids and NSAIDs within 1 year after treatment. Future investigations are recommended to explore factors influencing PD-prescribing trends and to assess patient satisfaction with PD treatment.

## Statement of authorship

Landon Trost: Conceptualization, Methodology, Investigation, Resources, Writing - Review & Editing, Funding Acquisition; Huan Huang: Conceptualization, Methodology, Investigation, Resources, Writing - Review & Editing, Funding Acquisition; Xu Han: Conceptualization, Methodology, Investigation, Resources, Writing - Review & Editing, Funding Acquisition; Chakkarin Burudpakdee: Conceptualization, Methodology, Investigation, Resources, Writing - Review & Editing, Funding Acquisition; Yiqun Hu: Conceptualization, Methodology, Investigation, Resources, Writing - Review & Editing, Funding Acquisition.
