# Supplementary material for: Treatment Patterns and Healthcare Outcomes with Collagenase Clostridium Histolyticum vs Surgery in Peyronie's Disease: A Retrospective Claims Database Analysis
Source: Sex Med. 2021 Mar 5;9(2):100321. doi: 10.1016/j.esxm.2021.100321 (PMC8072160; doi:10.1016/j.esxm.2021.100321)
Supplement: Supplementary Figures and Tables [file mmc1.pdf]

SUPPLEMENTAL TABLES

Supplemental Table 1: NDC and HCPCS Codes for Collagenase Clostridium Histolyticum

Xiaflex NDC Codes	66887000301, 66887000302
Xiaflex HCPCS Code	J0775

HCPCS = Healthcare Common Procedure Coding System; NDC = National Drug Code.

Supplemental Table 2: Diagnoses Codes for Peyronie's Disease

Diagnosis	ICD-9/ICD-10 Codes
Induration penis plastica	N48.6
Peyronie's disease	607.85

ICD = International Classification of Diseases.

Supplemental Table 3: Procedure Codes for Penile Surgeries of Interest

Procedure	ICD-9/ICD-10 Codes
Penile prosthesis implantation	0VUS0JZ; 0VUS4JZ, 0VUSXJZ, 64.95, 64.97
Plaque incision/excision	0VCSXZZ, 0VCS3ZZ, 0VCS0ZZ, 0VBSXZZ, 0VBS0ZZ, 0VBS3ZZ, 64.92, 64.2
Tunical plication	0VQS0ZZ, 64.49, 0VQS3ZZ, 0VQS4ZZ, 0VQSXZZ

ICD = International Classification of Diseases.

Supplemental Table 4: Diagnoses Codes for Clinical Events of Interest

Clinical Events of Interest	ICD-9/ICD-10 Codes
Dermatitis	692.89, L24.89, L25.9, L25.2, L23.4, L23.3, 692.2, L25.1, L24.9, L23.89, L23.5, L25.8, L25.3, L24.4, L24.0, L23.9, L24.5, 692.9, 692.4, 692.3, 692
Ecchymosis	R23.3, 782.7
Erectile dysfunction	N52.32, N52.9, 607.84, N52.8, N52.35, N52.31, N52.1, N52.03, N52.02, N52.33, N52.37, N52.34, N52.36, N52.39, 52.01, N52.2
Infection of the genitourinary tract	B37.42, A60.01, 98.3, 98.14, 95.8, 98.11, A54.01, A54.00, 98.33, 98.2, 98, 54.19, T83.61XS, A54.22, A54.21, A52.76, A51.0, 996.65, 98.32, 98.19, 98.1, A60.9, A60.00, A54.29, 98.34, 98.31, 91, 54.1, T83.61XA, A60.02, A54.23, 98.12, 98.13, 112.2, A54.09, A60.09, A60.1, B37.41, B37.49, T83.61XD, 54.13, A54.1, N48.21, N48.0, N48.29, A06.82, N47.6, N48.22, 607.1, 607.2
	607.81, N48.1
Paresthesias	782, R20.2
Penile injury	S30.21XA, S30.21XS, S30.21XD, 922.4
Penile injury due to foreign body	T83.82XD, 996.76, T83.82XA, T83.82XS
Penile foreign body	S31.22XD, S30.852D, S30.852A, T19.4XXS, S31.24XA, S31.24XS, S31.24XD, S30.852S, S31.22XS, 939.3, T19.4XXD, T19.4XXA, S31.22XA
Penile fracture	959.13, S39.840A, S39.840D, S39.840S

ICD = International Classification of Diseases.

Supplemental Table 5: Baseline Demographic and Clinical Characteristics Before and After PS-Matching

Measures	Before PS-matching			After PS-matching		
	CCH (N = 1227)	Surgery (N = 620)	*P*-value	CCH (N = 620)	Surgery (N = 620)	*P*-value
**Geographic region, n (%)**			.949			.887
Northeast	180 (14.7)	95 (15.3)		95 (15.3)	95 (15.3)	
Midwest	301 (24.5)	152 (24.5)		151 (24.4)	152 (24.5)	
South	624 (50.9)	316 (51)		320 (51.6)	316 (51)	
West	122 (9.9)	57 (9.2)		54 (8.7)	57 (9.2)	
**Payer type, n (%)**			.001			.329
Commercial	819 (66.7)	377 (60.8)		387 (62.4)	377 (60.8	
Medicare	4 (0.3)	3 (0.5)		1 (0.2)	3 (0.5%)	
Medicaid	8 (0.7)	17 (2.7)		6 (1)	17 (2.7)	
Self-insured*	383 (31.2)	220 (35.5)		222 (35.8)	220 (35.5)	
Other/Unknown	13 (1.1)	3 (0.5)		4 (0.6)	3 (0.5)	
**Plan type,† n (%)**			0.041			.065
HMO	81 (6.6)	49 (7.9)		44 (7.1)	49 (7.9)	
POS	54 (4.4)	27 (4.4)		27 (4.4)	27 (4.4)	
PPO	1056 (86.1)	538 (86.8)		544 (87.7)	538 (86.8)	
Indemnity	21 (1.7)	1 (0.2)		0 (0)	1 (0.2)	
Other/Unknown	15 (1.2)	5 (0.8)		5 (0.8)	5 (0.8)	

**Charlson Comorbidity Index**‡						
Mean (SD)	1.1 (1.7)	1.5 (2.0)	<.0001	1.4 (1.8)	1.5 (2.0)	.241
Median (IQR)	0 (0, 2)	1 (0, 2)		1 (0, 2)	1 (0, 2)	.207
Min	0	0		0	0	
Max	15	14		11	14	
Categories, n (%)			<.0001			.430
0	635 (51.8)	248 (40)		252 (40.6)	248 (40)	
1	268 (21.8)	146 (23.5)		158 (25.5)	146 (23.5)	
2	143 (11.7)	99 (16)		99 (16)	99 (16)	
3	80 (6.5)	55 (8.9)		44 (7.1)	55 (8.9)	
4+	101 (8.2)	72 (11.6)		67 (10.8)	72 (11.6)	
**Comorbidities, n (%)**						
Benign prostatic hyperplasia	441 (35.9)	219 (35.3)	.793	235 (37.9)	219 (35.3)	.344
Hypogonadism	327 (26.7)	188 (30.3)	.097	192 (31)	188 (30.3)	.806
Diabetes	264 (21.5)	157 (25.3)	.066	155 (25)	157 (25.3)	.894
Cardiovascular diseases	645 (52.6)	373 (60.2)	.002	372 (60)	373 (60.2)	.954
Ischemic heart disease	92 (7.5)	59 (9.5)	.135	63 (10.2)	59 (9.5)	.698
Heart failure	38 (3.1)	19 (3.1)	.970	28 (4.5)	19 (3.1)	.189
Stroke	61 (5)	53 (8.5)	.003	42 (6.8)	53 (8.5)	.227
Peripheral artery disease	41 (3.3)	28 (4.5)	.209	22 (3.5)	28 (4.5)	.396
Hypertension	595 (48.5)	352 (56.8)	.001	340 (54.8)	352 (56.8)	.493

Dyslipidemia	749 (61)	388 (62.6)	.521	402 (64.8)	388 (62.6)	.403
Dupuytren's contracture	50 (4.1)	7 (1.1)	.001	8 (1.3)	7 (1.1)	.763
Lower urinary tract symptoms	388 (31.6)	240 (38.7)	.002	233 (37.6)	240 (38.7)	.676
Prostate cancer	39 (3.2)	63 (10.2)	<.0001	37 (6)	63 (10.2)	.003
Radiation therapy§	4 (10.3)	10 (15.9)	.423	3 (8.1)	10 (15.9)	.157
Depression	227 (18.5)	129 (20.8)	.235	127 (20.5)	129 (20.8)	.887
Anxiety	249 (20.3)	132 (21.3)	.617	141 (22.7)	132 (21.3)	.526
Urolithiasis	109 (8.9)	55 (8.9)	.993	65 (10.5)	55 (8.9)	.331
Obesity	169 (13.8)	90 (14.5)	.664	83 (13.4)	90 (14.5)	.566
Penile trauma	10 (0.8)	11 (1.8)	.066	7 (1.1)	11 (1.8)	.346
Penile fracture	10 (0.8)	11 (1.8)	.066	7 (1.1)	11 (1.8)	.346
Other	0 (0)	1 (0.2)	.336	0 (0)	1 (0.2)	>.99
Neurological conditions leading to ED	449 (36.6)	228 (36.8)	.939	251 (40.5)	228 (36.8)	.172
Spinal cord injury	2 (0.2)	2 (0.3)	.606	1 (0.2)	2 (0.3)	.564
Lower back pain	449 (36.6)	228 (36.8)	.939	251 (40.5)	228 (36.8)	.172
Motor vehicle injury	0 (0)	1 (0.2)	.336	0 (0)	1 (0.2)	
**History of radical prostatectomy, n (%)**	12 (1)	32 (5.2)	<.0001	12 (1.9)	32 (5.2)	.001
**Penile events of Interest, n (%)**						
Corporeal rupture	10 (0.8)	11 (1.8)	.066	7 (1.1)	11 (1.8)	.346
ED	632 (51.5)	430 (69.4)	<.0001	423 (68.2)	430 (69.4)	.620

Foreign body/allergic reaction	129 (10.5)	74 (11.9)	.356	69 (11.1)	74 (11.9%)	.662
Paresthesia	137 (11.2)	78 (12.6)	.371	85 (13.7)	78 (12.6)	.558
Penile ecchymosis	6 (0.5)	1 (0.2)	.435	4 (0.6)	1 (0.2)	.180
Penile fibrosis	1 (0.1)	1 (0.2)	>.99	1 (0.2)	1 (0.2)	>.99
Penile hematoma	5 (0.4)	2 (0.3)	>.99	3 (0.5)	2 (0.3)	.655
Penile infection	11 (0.9)	8 (1.3)	.428	5 (0.8)	8 (1.3)	.405
Penile inflammation	34 (2.8)	19 (3.1)	.721	17 (2.7)	19 (3.1)	.732
Penile narrowing	11 (0.9)	8 (1.3)	.428	7 (1.1)	8 (1.3)	.796
Penile pain	142 (11.6)	126 (20.3)	<.0001	107 (17.3)	126 (20.3)	.144
Penile swelling	35 (2.9)	27 (4.4)	.091	23 (3.7)	27 (4.4)	.564
Penile/blood blisters	0 (0)	0 (0)	NA	0 (0)	0 (0)	NA
Phimosis/ paraphimosis	9 (0.7)	7 (1.1)	.386	6 (1)	7 (1.1)	.782
Suture granuloma	2 (0.2)	3 (0.5)	.342	2 (0.3)	3 (0.5)	.655
Urethral injury	2 (0.2)	1 (0.2)	>.99	2 (0.3)	1 (0.2)	.564
**Time from earliest PD diagnosis to index (months)**						
Mean (SD)	11.9 (12.7)	11.6 (13.3)	.716	12.4 (12.7)	11.6 (13.3)	.325
Median (IQR)	7.5 (3.0, 15.8)	7.0 (2.8, 14.7)	.246	7.9 (3.3, 17.0)	7.0 (2.8, 14.7)	.198
Min	0.2	0.0		0.3	0.0	
Max	77.5	84.1		74.0	84.1	

Categories, months, n (%)			.559			.158
≤ 6	517 (42.1)	280 (45.2)		249 (40.2)	280 (45.2)	
>6 and ≤12	294 (24)	144 (23.2)		143 (23.1)	144 (23.2)	
>12 and ≤24	256 (20.9)	115 (18.5)		143 (23.1)	115 (18.5)	
>24	160 (13)	81 (13.1)		85 (13.7)	81 (13.1)	

[*]Self-insured plan is where the employer takes the financial risk for providing health care benefit to its employees.

[†]HMO coverage is historically associated with lower premiums, relatively modest co-payments and deductibles, and tightest provider network management controls. Indemnity insurance is traditional health insurance, typically with the highest premiums and the greatest choice of providers. Patients typically pay 20% coinsurance rates and higher deductibles than those in HMO plans for select services. POS plans are a hybrid of HMO and Indemnity plans. If a patient stays within a provider network and does not self-refer, patient contributions are structured like an HMO. If the patient elects to self-refer, contributions are structured like Indemnity plans. PPO plans are structured like Indemnity plans, but the fees paid to providers are discounted if the patient stays within the network.

[‡]Calculated from ICD-9-CM/ICD-10-CM codes in any position during the baseline period. Because the objective of the comorbidity score is to evaluate underlying comorbidity burden independent of the cancer, ICD diagnosis codes for cancer were excluded from the Charlson Comorbidity Index score calculation.

[§]Radiation therapy was measured among patients with prostate cancer during the baseline period.

CCH = collagenase clostridium histolyticum; ED = erectile dysfunction; HMO = Health Maintenance Organization; ICD = International Classification of Diseases; IQR = interquartile range; POS = point of service; PPO = preferred provider organization; PS = propensity score; SD = standard deviation.

Supplemental Table 6: Treatment History Before and After PS-Matching

Measures	Before PS-matching			After PS-matching		
	CCH (N = 1227)	Surgery (N = 620)	*P*-value	CCH (N = 620)	Surgery (N = 620)	*P*-value
Treatment of PD, n (%)						
Analgesics	852 (69.4)	474 (76.5)	.002	458 (73.9)	474 (76.5)	.294
Prescription non-steroidal anti-inflammatory drugs	493 (40.2)	274 (44.2)	.098	265 (42.7)	274 (44.2)	.602
Opioids	752 (61.3)	428 (69)	.001	411 (66.3)	428 (69)	.296
Oral therapy	368 (30)	142 (22.9)	.001	148 (23.9)	142 (22.9)	.682
Potaba	11 (0.9)	6 (1)	.880	5 (0.8)	6 (1)	.763
Tamoxifen	3 (0.2)	0 (0)	.555	1 (0.2)	0 (0)	>.99
Colchicine	84 (6.8)	29 (4.7)	.066	32 (5.2)	29 (4.7)	.701
Carnitine	1 (0.1)	0 (0)	>.99	0 (0)	0 (0)	NA
Pentoxifylline	280 (22.8)	106 (17.1)	.004	115 (18.5)	106 (17.1)	.508
Procarbazine	0 (0)	0 (0)	NA	0 (0)	0 (0)	NA
Prescribed omega-3 fatty acids	23 (1.9)	18 (2.9)	.157	11 (1.8)	18 (2.9)	.194
Prescribed co-enzyme Q10	0 (0)	0 (0)	NA	0 (0)	0 (0)	NA
Corticosteroid injections	499 (40.7)	264 (42.6	.431	263 (42.4)	264 (42.6)	.956

Intralesional injection therapy	504 (41.1)	267 (43.1)	.413	264 (42.6)	267 (43.1)	.870
Verapamil	8 (0.7)	2 (0.3)	.510	4 (0.6)	2 (0.3)	.414
Interferon alpha-2B	2 (0.2)	2 (0.3)	.606	1 (0.2)	2 (0.3)	.564
Vacuum erection device	42 (3.4)	45 (7.3)	0.000	32 (5.2)	45 (7.3)	.123
Treatment of ED, n (%)						
PDE-5 inhibitors	269 (21.9)	164 (26.5)	.030	165 (26.6)	164 (26.5)	.945
Alprostadil	95 (7.7)	70 (11.3)	.012	58 (9.4)	70 (11.3)	.277
Testosterone	208 (17)	110 (17.7)	.671	121 (19.5)	110 (17.7)	.431
Treatment of BPH, n (%)						
Alpha blockers	15 (1.2)	11 (1.8)	.342	12 (1.9)	11 (1.8)	.827
5-Alpha reductase inhibitors	59 (4.8)	29 (4.7)	.901	31 (5)	29 (4.7)	.786
TURP	5 (0.4)	1 (0.2)	.670	3 (0.5)	1 (0.2)	.317
HoLEP	0 (0)	0 (0)	NA	0 (0)	0 (0)	NA
TUMT	0 (0)	0 (0)	NA	0 (0)	0 (0)	NA
TUIP	0 (0)	0 (0)	NA	0 (0)	0 (0)	NA
BPH thermotherapy	0 (0)	1 (0.2)	.336	0 (0)	1 (0.2)	>.99
TUNA	0 (0)	1 (0.2)	.336	0 (0)	1 (0.2)	>.99
Laser therapy for BPH	3 (0.2)	5 (0.8)	.128	3 (0.5)	5 (0.8)	0.414
PUL	0 (0)	1 (0.2)	.336	0 (0)	1 (0.2)	>.99

BPH = benign prostatic hyperplasia; CCH = collagenase clostridium histolyticum; ED = erectile dysfunction; HoLEP = holmium laser enucleation of the prostate; NA = not applicable; PD = Peyronie's disease; PDE-5 = phosphodiesterase-5; PS = propensity score; PUL = prostatic urethral lift; TUMT = transurethral microwave therapy; TUNA = transurethral needle ablation; TUIP = transurethral incision of the prostate; TURP = transurethral resection of the prostate.

Supplemental Table 7: Post-procedural Penile-Related Complications During the 12-Month Post-Index Period (PS-Matched Cohorts)

	CCH cohort (N = 620) n (%)	Surgery cohort (N = 620) n (%)	*P*-value
Any penile event of interest*, N (%)	323 (52.1)	466 (75.2)	<.0001
Corporeal rupture	11 (1.8)	5 (0.8)	.134
Erectile dysfunction	278 (44.8)	403 (65.0)	<.0001
Foreign body/allergic reaction	7 (1.1)	22 (3.5)	.005
Paresthesia	12 (1.9)	20 (3.2)	.157
Penile ecchymosis	0 (0)	1 (0.2)	.317
Penile fibrosis	1 (0.2)	5 (0.8)	.103
Penile hematoma	7 (1.1)	1 (0.2)	.034
Penile infection	0 (0)	9 (1.5)	.003
Penile inflammation	14 (2.3)	24 (3.9)	.105
Penile narrowing	3 (0.5)	7 (1.1)	.206
Penile pain	55 (8.9)	111 (17.9)	<.0001
Penile swelling	32 (5.2)	50 (8.1)	.044
Penile/blood blisters	0 (0)	0 (0)	-
Phimosis/paraphimosis	4 (0.6)	17 (2.7)	.005
Suture granuloma	1 (0.2)	2 (0.3)	.564
Urethral injury	0 (0)	2 (0.3)	.157

*Penile event included any event present during the 12-month post-index period regardless of the presence during the pre-index period. Separate data are available for newly reported post-procedural complications which were absent during pre-index period.

CCH = collagenase clostridium histolyticum; PS = propensity score.

SUPPLEMENTAL FIGURES

Supplemental Figure 1. Cohort for the treatment trend analysis.

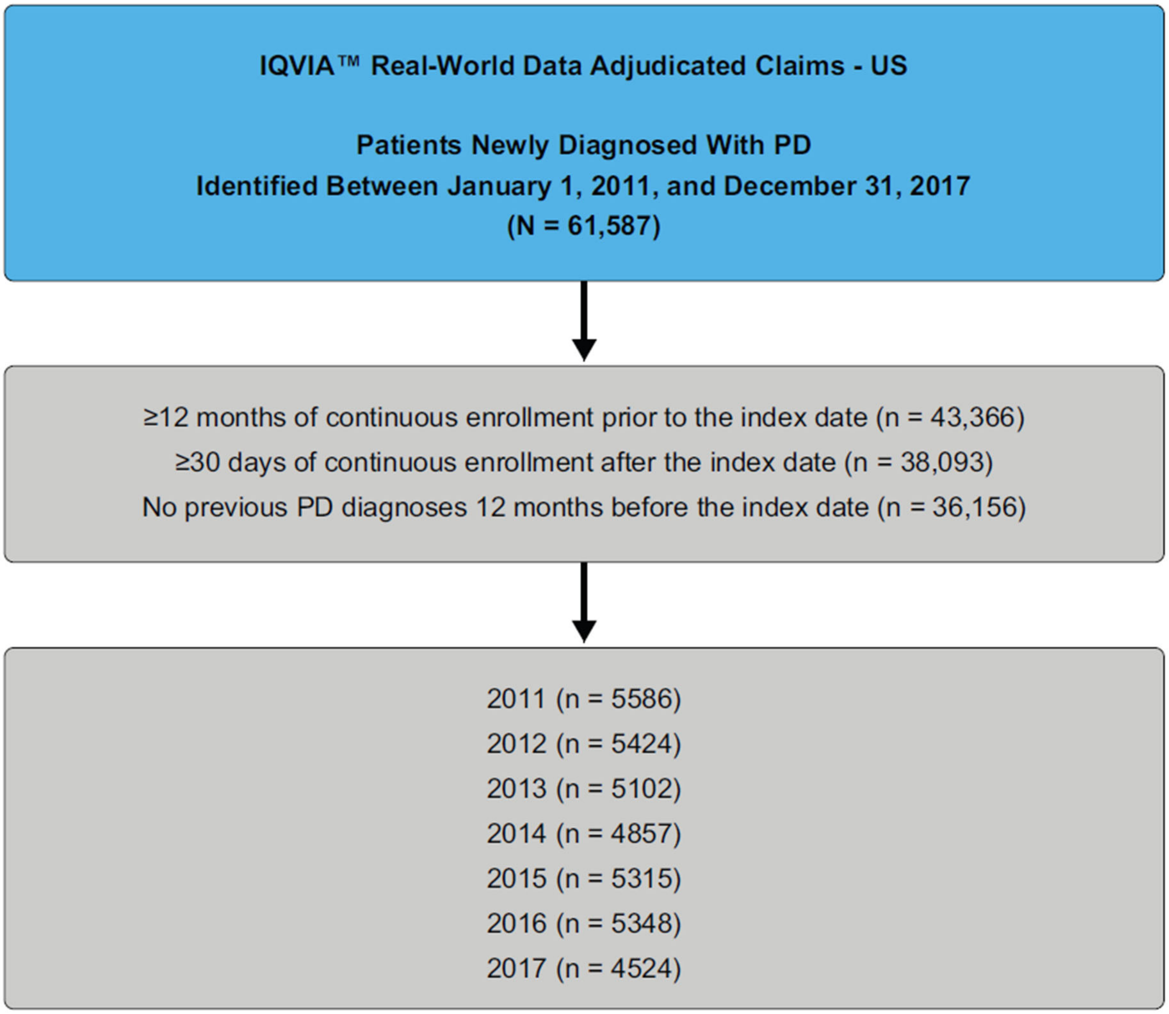

Note: index date is the date of the first PD diagnosis.

PD = Peyronie's disease.

Supplemental Figure 2. Study schema (matched and unmatched cohorts).

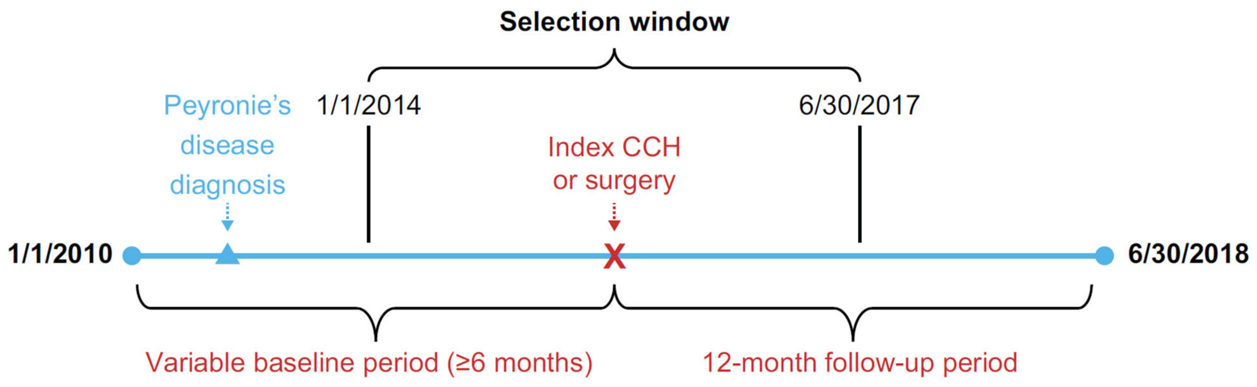

Note: Index date = first CCH or surgery treatment within the selection period.

CCH = collagenase clostridium histolyticum.
